# Neuroticism in Young Women with Fibromyalgia Links to Key Clinical Features

**DOI:** 10.1155/2012/730741

**Published:** 2012-02-19

**Authors:** Katrina Malin, Geoffrey Owen Littlejohn

**Affiliations:** ^1^Faculty of Medicine, Monash University, Melbourne, VIC 3168, Australia; ^2^Department of Rheumatology, Monash Medical Centre, Melbourne, VIC 3168, Australia

## Abstract

*Objective*. We examined personality traits in young women with FM, in order to seek associations with key psychological processes and clinical symptoms. *Methods*. Twenty-seven women with FM and 29 age-matched female healthy controls [HC] completed a series of questionnaires examining FM symptoms, personality and psychological variables. *Results*. Significant differences between characteristic FM symptoms (sleep, pain, fatigue, and confusion) as well as for the psychological variables of depression, anxiety, and stress were found between FM and HC (*P* < 0.001). Neuroticism was the only subscale of the Big Five Inventory that showed a significant difference between the FM group and HC group [*P* < 0.05]. Within the FM group, there was a significant association between the level of the neuroticism and each of pain, sleep, fatigue, and confusion, depression, anxiety, and stress (*P* < 0.05–0.01). The association between the level of neuroticism and the level of stress was the strongest of all variables tested (*P* < 0.001). *Conclusion*. The personality trait of neuroticism significantly associates with the key FM characteristics of pain, sleep, fatigue and confusion as well as the common co-morbidities of depression, anxiety and stress. Personality appears to be an important modulator of FM clinical symptoms.

## 1. Introduction

Fibromyalgia (FM) is a common musculoskeletal pain syndrome, characterized by widespread pain and abnormal tenderness, associated with variable stiffness, fatigue, poor quality sleep, cognitive disturbance, and emotional distress. It is found more commonly in women than men (9 : 1, resp.) and reported to be present in around 2–5% of the female population within Western societies [1, 2]. As a result of the key FM symptoms of pain, fatigue, sleep disturbance, and confusion, major quality of life issues arise that have high societal impact, including medical, psychological, and financial [3].

The symptoms of FM relate to disturbed central processing of pain-related and other neural functions [4]. These in turn are modulated by changes in downward control originating in emotion-related brain areas, such as the anterior cingulate cortex and limbic system [5]. Psychological factors, including inadequate coping with external stressors, are common in FM [6]. From clinical observation, FM patients often appear to have personality styles that allow for easy translation of life events into emotional distress. For instance, patients with FM are said to be demanding [7], perfectionist, and have high expectations of themselves and also the people around them [8]. However, although previously suggested no specific FM personality type has been defined [7]. Despite this, personality is an important filter that modulates a person's coping response to psychological stressors [9] and may facilitate translation of these stressors to physiological responses that drive the FM mechanism. Thus aspects of personality style may modulate the central sensitization mechanism that underlies maintenance and/or exacerbation of symptoms of FM.

The traits of extraversion and neuroticism have been widely studied as potential mechanisms that may underlie certain illnesses [10, 11]. In particular, neuroticism, defining an enduring tendency to experience negative emotional states, has been associated with symptoms of depression, anxiety and high arousal. Patients with migraine, pain, chronic fatigue, and irritable bowel all score significantly higher on the neuroticism scale compared to healthy controls. These findings may be biased by health seeking behaviour as it itself has also been linked to neuroticism [12]. There also have been a number of other studies that suggest a causal effect between neuroticism and health. Pain mechanisms are heighted by neuroticism reflecting that psychosocial factors influence biological mechanisms [12]. Furthermore, both neuroticism and extraversion have been associated with the regulation of the autonomic nervous system and pain responses [13]. Neuroticism will also influence recall and in turn memory and is an issue pertinent to patients with FM [14].

Limited and inconsistent [13] studies have investigated the role of personality in the development, maintenance, and exacerbation of symptoms associated with FM. Previous studies focussed on personality using scales based on levels of psychopathology, such as the Minnesota Multiphasic Personality Inventory [MMPI I/2], or take a psychobiological/biological relevance approach using scales such as the Temperament and Character Inventory (TCI) or Karolinska scale of personality [15]. It has been shown that high levels of neuroticism link to increased perception of pain and other symptoms associated with FM [16, 17]. This may occur through the effect of personality on the type of coping techniques used to deal with pain rather than a direct effect on the pain itself [12, 17].

The key domains contributing to the clinical phenotype of FM, namely, pain, poor sleep, fatigue, cognitive dysfunction (confusion), emotional distress (anxiety and depression), and stress are well defined within the American College of Rheumatology [ACR] 1990 classification [18] and the 2010 diagnostic [19] criteria for FM. We aimed to explore whether personality traits influence these characteristic symptoms of FM. We surveyed young women with FM in order to minimize long-term secondary and adaptive effects of the condition on these FM outcomes. We specifically wished to see if the characteristic FM symptoms of pain, sleep, confusion, depression, anxiety, and stress associate with personality traits and if so how these factors might interlink.

## 2. Methods

### 2.1. Ethics

Ethics approval was obtained through relevant committees of Monash University and Monash Medical Centre, Melbourne, Australia.

### 2.2. Subjects

The participants in this study consisted of volunteer women who were sourced from a variety of areas including a FM self-management program, notices in local newspapers, a fibromyalgia treatment clinic, and local rheumatologists. Twenty-five female FM patients fulfilling ACR 1990 classification criteria and 27 female HCs, all healthy individuals with no pain condition and recruited by word of mouth were identified. All were under age of 39 years of age.

### 2.3. Procedures

All participants were sent written information regarding the study along with a consent form which, when signed, was followed by a series of questionnaires. These included the Fibromyalgia Impact Questionnaire [20], the Profile of Mood States [21], and the Perceived Stress Scale [22]. Participating FM women were also contacted 12 months later to complete the same battery of questionnaires, with a response rate of 56%.

### 2.4. Instruments

The following instruments were applied to all FM and HC subjects at time zero and to 56% of FM patients 12 months later.

1. The Big 5 Personality Inventory (BFI) [11]: a validated 44-item personality scale, scored as 1 (disagree strongly) through 5 (agree strongly) to indicate the extent of agreement with the items. The 44 items comprise 5 subscales of extraversion, agreeableness, conscientiousness, neuroticism, and openness.
2. Fibromyalgia Impact Questionnaire (FIQ) [20]: a validated 20-item functional ability questionnaire, which measures how an individual's symptom characteristics impact their daily functioning for the preceding week. Individual subscales include sleep, depression, anxiety, and pain and use a 0 to 10 cm visual analogue scale (VAS), measuring left of line for “no impact of subscale” through to the far right, “worst possible impact”.
3. Perceived Stress Scale (PSS) [22]: a validated scale that assesses the degree an individual experiences feelings of being overwhelmed by stressful life events over the past month. The scale is a 10-item, 5-point likert scale ranging from 0 (never) to 4 (very often) with scores ranging from 0 to 40.
4. Profile of Mood States (POMS) [21] confusion subscale: a validated scale that measures individual aspects of mood as well as a total overall mood score. The POMS identifies adjective words that describe feelings that are indicative of mood states. The questionnaire asks individuals to rate on a scale from zero (not at all) to four (extremely) which best describes how they have felt over the past week. The scale includes a total of 65 definitions that represent the 6 subscales that include Tension-Anxiety, Depression-Dejection, Anger-Hostility, Vigour, Fatigue, and Confusion. A total mood score is obtained by summing all subscale scores with vigour inversed. The subscale of confusion was used to represent the cognitive dysfunction seen in FM. The single word items that reflect confusion include bewildered, confused, unable to concentrate, forgetful, uncertain, and efficient (score reversed).

### 2.5. Statistical Analysis

Initial descriptive analysis was conducted, along with normality checks, using SPSS (PASW version). Chi-squared test was used to test for differences in group demographics. *t*-Tests, means and standard deviations were used to explore the differences between groups in symptom characteristics and stability of personality traits. ANOVAs were performed to compare the differences between the groups that explored levels of neuroticism (high, medium, and low) for symptom characteristics of FM within the FM group. Bivariate (Pearson) correlation was used to compare the relationships between the variables of FM and levels of neuroticism.

## 3. Results

The demographic characteristics of the total FM group and the HC group at time zero are shown in Table 1. The groups were matched for age (*P* = 0.1). At both time zero and at 12 months followup the FM group reported that 100% were married and the majority were working part time in either semi- or fully professional roles, holding tertiary qualifications with income levels around $AUD 20,000. Compared to HCs more FM patients were part-time workers and had lower mean incomes. 

Table 2 shows the personality traits in the FM subgroup at the two time periods of the study, time zero and 12 months later. There was stability within the subscales over 12 months with the exception of the subscale of agreeableness. This confirms previous research that shows that the BFI operational definition of neuroticism is robust over time in this FM population.

Table 3 details symptom characteristics and mean personality traits in FM and HC groups. As expected, all the symptom characteristics traditionally associated with FM were found to be significantly different between the FM and HC groups. Sleep and fatigue were rated higher than pain on the VAS scales in both groups. In contrast, the subscales of personality showed a significant difference only for the scale of neuroticism.

 The relationship between neuroticism and the symptoms that are associated with FM is shown in Table 4. It is evident that most symptoms of FM are significantly influenced by neuroticism in the FM group with the exception of pain and confusion. The correlation showed moderate-to-strong positive relationships suggesting a linear relationship that as the level of neuroticism increases so does the level of FM symptoms. There are strong associations between neuroticism and stress and anxiety and a relatively strong relationship between neuroticism and depression.

The association between neuroticism and FM symptoms was explored further, by equally separating for low, medium, and high levels of neuroticism, as shown in Table 5. Significant differences were found between all high and low neuroticism groups for each of the characteristic symptoms of FM, with the exception of stress. There were statistically significant differences between all three levels of neuroticism, high, medium, and low, and stress, indicating a gradient effect of neuroticism severity and level of stress. In contrast, a ceiling effect was found for pain, with the group that had a medium level of neuroticism reporting a higher mean for pain than the group categorised as having high levels of neuroticism. It is noted that even with the high levels of neuroticism the levels of depression and anxiety would only be clinically rated as moderate. Higher levels of fatigue and worsening quality of sleep were present as the level of neuroticism increased.

## 4. Discussion

The cause for fibromyalgia remains unclear. However, increased sensitivity and responsiveness of pain-related neural systems in the spinal cord and brain has been shown through numerous studies [23]. This change in spinal cord dorsal horn neurone function allows innocuous stimuli to access the pain system and cause pain responses in the brain. Low threshold mechanoreceptors, associated with muscle and joint tone and movement, for instance, provide significant sensory input to facilitate this process [24]. The spinal cord dorsal horn pain-related neuron sensitivity in turn is influenced by downward control mechanisms from the midbrain and from the brain itself. These involve, in particular, the emotion linked regions of the brain such as the frontal cortex, the anterior cingulate gyrus, the amygdale, and other limbic structures [5, 25, 26].

This “top-down” model fits with the biopsychosocial model of fibromyalgia [27]. Central psychological factors, acting under the influence of external social stressors, result in emotional distress that in turn may activate physiological stress responses if modifying “filters” allow. These filters include the individual's personality, belief system, sleep pattern, and other psychosocial factors; each possibly facilitating or impeding outcomes. Patients with fibromyalgia have been characterised as having significant background psychological distress [28]. Increased activity of the autonomic nervous system is present in many works [29]. In others there is evidence of poor adaptation to routine stressors with increased rates of catastrophization and poor coping skills indicating a link between intrinsic stress coping styles and inputs from the outside world [30].

In this study, we were interested in the role of personality in this process. Personality has been difficult to be clearly defined and many different systems and approaches have been used to try to better define the essential elements of personality [31]. We hypothesize that certain personality styles are able to influence interpretation of everyday stressors and either blunt or amplify subsequent physiological responses in the brain. We thus see personality as a modifying filter between external stressors and subsequent physiological responses, particularly those that relate the emotional part of the brain to the downward control systems influencing the spinal cord sensory sensitivity centres. Hence, we see a link between psychological influences and the clinical features of fibromyalgia, as expressed by the phenotype of widespread pain, widespread tenderness, dermatographia, muscle stiffness, and related clinical events.

There are numerous variables that might influence stress reactivity in a fibromyalgia population. The characteristics of the condition itself, with resultant pain, fatigue, sleep disturbance, and significant disability, will cause stress and other feedback events in its own right. In order to lessen these effects we chose young female patients in order to define a population less influenced by age and accumulative life stress factors.

The clinical characteristics of our fibromyalgia population, when compared to healthy controls, showed typical scores on visual analogue scales for the domains of pain, sleep, fatigue, depression, and anxiety. In our fibromyalgia population we found the depression and anxiety scores to be around 4.7 out of 10 suggesting that this group had only moderate levels of mood disturbance. We also found that in this group sleep and fatigue abnormalities rated higher than pain.

In the study we used the big 5 personality inventory that examines traits linked to different personality styles. There are very limited studies into personality traits and FM that have used the big 5 inventory, however, neuroticism and to a lesser extent extraversion have been examined through use of other personality measures [17]. Personality is generally considered to be a consistent collection of affective, cognitive, and behavioural patterns. Underlying traits or characteristics represent both the similarities and the differences that typify the individual together with how, when, and why people adapt within certain environments. One approach to personality is a trait perspective which is reflected in the components of the “The big 5” (BFI). This instrument is a profile of five hierarchical descriptors that are said to capture a broad level of concepts and commonalities among most of the existing systems of personality description and hence it provides an integrative model for personality research [10, 11]. These 5 traits are defined by descriptors presented on a continuum. In the BFI neuroticism is defined in terms of “is depressed, worries a lot, can be moody, gets nervous easily, and remains calm (scored in reverse)”, all statements that reflect elements of anxiety. However, the descriptors also include “becoming upset, tense, worried, and not relaxed.” A number of studies show links between development and exacerbation of chronic health conditions in general and the personality style defined as neuroticism, with a linear increase of different pathologies to increasing neuroticism [12].

We show that the BFI is stable over time in this fibromyalgia population, with the exception of the trait of agreeableness, which unlike other traits, did show a significant difference between patients examined over a twelve-month period. The other four characteristics of the big 5 scale, that is, neuroticism, extraversion, openness, and conscientiousness, showed no significant difference over the twelve-month period. When the age-matched fibromyalgia group was compared to a group of healthy pain-free controls it was noted that only neuroticism was significantly different between the two groups. This observation supports previous findings that fibromyalgia subjects have more neurotic traits than healthy controls [32].

In this current study of younger female FM patients there was no significant relationship in the total group between neuroticism and pain or confusion. Although this might suggest there is indeed no relationship we feel it is more likely that this reflects methodology of assessment and ceiling effects for these variables. Additionally, potential confounding variables may be impacting on this relationship, such as control, and these require more detailed investigation. There was however positive moderate-to-strong correlations between neuroticism and fatigue, sleep, depression, anxiety, and stress, all key symptoms of FM.

When levels of neuroticism were arbitrarily classified as low, medium, or high, there was, in general, significant correlation between moderate and higher levels of neuroticism and FM symptoms compared to lower levels. There was less variation between the mid-to-high levels of neuroticism than there was between the low-to-mid levels of neuroticism. For instance, for pain a ceiling effect was present with no statistically significant difference between pain in the moderate-to-high levels of neurotic traits. Stress was a striking exception, being significantly different between all levels of neuroticism and showing a clear gradient effect with severity of FM symptoms. Stress has been noted to be a major factor in the exacerbation and potential development of FM [6]. Thus as neuroticism traits increase in the patient with FM the more symptoms that patient will experience. As previously stated, the neuroticism trait as defined by the Big 5 inventory is based on questions that, in many instances, link to how an individual perceives and reacts to stress. Hence there is some commonality in components of the various instruments used to measure, for instance neuroticism, stress, or anxiety. While this may account for some of the positive associations between these features it is felt that the trait defined as neuroticism is a well-accepted and stand-alone entity that defines a certain personality style. As such the trait in its own right appears as an important component of FM.

The pain, sleep change, and fatigue of FM may be further aggravated by pain-associated maladaptive coping techniques and negative pain beliefs, each also likely influenced by personality. With such interacting feedback influencing on the FM process it is therefore a challenge to identify how exactly personality impacts on fibromyalgia. This is further complicated by the multifaceted nature of personality with no simple assessment tool accounting for all its aspects.

Personality has been suggested to be a factor that increases the risk for the development of FM [9, 33–35]. In this study personality, specifically neuroticism was found to modulate symptoms associated with FM, in particular stress levels. Neuroticism also modulated sleep quality and fatigue. Thus with increased levels of neuroticism the individual has poorer quality of sleep, higher levels of fatigue, and increased levels of stress.

As this is a cross-sectional study only limited associations can be drawn from this analysis. In addition, the duration of pain was not part of the analysis and hence any conclusions that potentially associate being younger with less influence of neuroticism need to be taken cautiously. However, it is possible that being younger will equate to less time to become entrenched in behaviours that reinforce factors that in turn further contribute to FM [36]. There are no other studies on neuroticism in young females with FM.

A number of demographic differences between the FM group and the HC group might imply that there are a number of psychosocial variables that may influence the findings. Past studies have noted that often the participants that enrol in such studies are not reflective of the general population of FM [37] indicating caution in generalizing these findings to the total FM population. The fact that many of these participants had been involved in previous treatment programs suggests that this group potentially may be more proactive and this could be reflected in their personality style. Additionally this study needs to be replicated in an older age population.

In this study we found that certain personality styles, specifically neuroticism, associate with clinical features of FM. It is proposed therefore that personality is an important filter that modulates a person's response to psychological stressors and is involved in translation of these stressors to physiological responses driving the fibromyalgia mechanism. This area of research is complex but important to the understanding of fibromyalgia, especially from a biopsychosocial approach that integrates mind and body.

## Figures and Tables

**Table 1:** Demographic characteristics of fibromyalgia (FM) and healthy control (HC) groups.

	FM	HC	Significance
	(*N* = 25)	**(** *N* = 27**)**	(Chi-square)
Age: years			NS
20–29	8	15	
30–39	17	12	
Marital:			NS
Single	4	9	
Married	14	10	
Other	7	8	
Occupation:			NS
Semiprofessional	8	1	
Professional	8	16	
Other	7	9	
Work Status			NS
Fulltime	8	18	
Part time	11	8	
Casual	1	1	
Education			*P* < 0.01
Tertiary	12	22	
College	8	0	
Other	5	5	
Average Income (Australian Dollar)	<$20,000	$41–60,000	*P* < 0.05

**Table 2:** Paired Sample *t*-Statistics for personality traits in fibromyalgia patients over 12-month time period.

	*t*	*P*
Personality trait:		
Extraversion	0.24	N/S
Neuroticism	1.66	N/S
Agreeableness	2.84	0.05
Conscientiousness	1.33	N/S
Openness	0.81	N/S

**Table 3:** Independent *t*-tests between fibromyalgia patients (FM) and healthy controls (HC) for symptom characteristics and personality traits (means ± standard deviation).

	FM	HC	*t*	df	*P*
Symptom Characteristic					
Pain	6.36 ± 2.25	0.17 ± 0.10	13.18	47	0.000
Fatigue	8.52 ± 1.33	2.88 ± 2.44	10.16	48	0.000
Sleep	8.24 ± 1.67	3.33 ± 2.75	7.71	50	0.000
Confusion	11.0 ± 4.45	6.19 ± 5.87	3.31	50	0.01
Depression	4.72 ± 2.49	1.41 ± 2.56	4.72	50	0.000
Anxiety	4.76 ± 3.07	1.74 ± 2.16	4.13	50	0.000
Stress	30.16 ± 5.89	24.15 ± 7.30	3.25	50	0.01
Personality traits					
Extraversion	24.35 ± 6.29	27.43 ± 5.84	−1.68	50	NS
Neuroticism	25.95 ± 5.22	23.91 ± 6.04	2.36	50	0.05
Agreeableness	35.21 ± 3.94	33.57 ± 4.03	1.07	50	NS
Conscientiousness	35.10 ± 4.24	33.29 ± 5.44	1.20	50	NS
Openness	35.20 ± 5.99	34.43 ± 1.02	0.44	50	NS

NS: not significant.

**Table 4:** Relationships between neuroticism and symptom characteristics in fibromyalgia group.

	Neuroticism	
	*r*	*P*
Symptom Characteristics:		
Pain	0.00	NS
Fatigue	0.47	0.05
Sleep	0.40	0.05
Confusion	0.29	NS
Depression	0.58	0.01
Anxiety	0.63	0.001
Stress	0.75	0.000

NS: not significant.

**Table 5:** ANOVA results for levels of the neuroticism trait of low (1), medium (2), and high (3) according to the fibromyalgia group individual symptom characteristic.

				Neuroticism level		
	*F*	df	*P*	Mean ± SD	Groups	*P*
Symptom						
Pain:	4.48	2, 46	0.05	(1) 4.75 ± 1.39	1-2	0.05
				(2) 6.80 ± 2.14	1–3	0.05
				(3) 6.28 ± 2.11		
Sleep:	7.64	2, 49	0.001	(1) 3.31 ± 3.07	1-2	0.05
				(2) 6.37 ± 2.87	1–3	0.01
				(3) 7.18 ± 3.05		
Fatigue:	7.10	2, 47	0.002	(1) 3.31 ± 2.98	1-2	0.01
				(2) 6.65 ± 3.20	1–3	0.01
				(3) 7.00 ± 3.06		
Confusion:	6.44	2, 49	0.003	(1) 4.63 ± 3.46	1–3	0.01
				(2) 10.05 ± 6.11	1-2	0.01
				(3) 10.41 ± 5.44		
Depression:	14.60	2, 49	0.001	(1) 0.44 ± 0.89	1-2	0.01
				(2) 3.42 ± 2.99	1–3	0.000
				(3) 4.94 ± 2.70		
Anxiety:	12.47	2, 49	0.000	(1) 0.88 ± 1.09	1-2	0.05
				(2) 3.32 ± 2.83	1–3	0.000
				(3) 5.34 ± 3.03		
Stress:	25.67	2, 49	0.000	(1) 19.88 ± 4.16	1-2	0.000
				(2) 2. 28.05 ± 5.62	1–3	0.000
				(3) 3. 32.65 ± 5.50	2-3	0.05
